# Supplementary material for: Supply network position, digital transformation and innovation performance: Evidence from listed Chinese manufacturing firms
Source: PLoS One. 2022 Dec 15;17(12):e0279133. doi: 10.1371/journal.pone.0279133 (PMC9754206; doi:10.1371/journal.pone.0279133)
Supplement: Attachment — Submitted filename: PONE-D-22-18835_reviewer_28.07.22.pdf [file pone.0279133.s002.pdf]

PLOS ONE

Supply network position, digital transformation and innovation performance: evidence from listed Chinese manufacturing firms

--Manuscript Draft--

Manuscript Number:	PONE-D-22-18835
Article Type:	Research Article
Full Title:	Supply network position, digital transformation and innovation performance: evidence from listed Chinese manufacturing firms
Short Title:	Supply network position, digital transformation and innovation performance
Corresponding Author:	chunyan du<br>Yunnan University of Finance and Economics<br>Kunming, CHINA
Keywords:	supply network; centrality; structural hole; innovation performance; digital transformation
Abstract:	This study provides evidence for the supply network position influencing innovation performance and the moderating effect of digital transformation. Supply chain relationships have been evaluated in earlier research to demonstrate how concentrations of customers and suppliers may either favorably or adversely impact innovation. These metrics, however, only take into account how closely a firm is connected to its direct customers or suppliers. This study integrates the top five suppliers and customers of Chinese listed manufacturing firms and considers the relationship embeddedness of each firm's direct customers and suppliers, as well as the structure embeddedness among the customers' customers, customers' suppliers, suppliers ' customers, and suppliers ' suppliers to reveal the true impact of supply chain relationships on innovation performance.The top five suppliers and consumers of each firm are chosen to build a supply network for each year using panel data of listed Chinese manufacturing firms from 2013 to 2020. Social network analysis is used to determine network centrality and structural holes.The results show that in the supply network, network centrality and structural holes are significantly negatively correlated with innovation performance, especially in large firms, non-state-owned firms and firms in recession phase. According to the moderating effect model, digital transformation is an efficient way for declining the negative effect of supply network position on innovation performance. The research results will further improve the supply network cooperation mechanism, which is of great significance for improving supply chain resilience and firms' innovation.
Order of Authors:	Chunyan Du
	Qiang Zhang
Additional Information:	
Question	Response
**Financial Disclosure**<br><br>Enter a financial disclosure statement that describes the sources of funding for the work included in this submission. Review the submission guidelines for detailed requirements. View published research articles from *PLOS ONE* for specific examples.<br><br>This statement is required for submission and **will appear in the published article** if the submission is accepted. Please make	The funders had no role in study design, data collection and analysis, decision to publish, or preparation of the manuscript

Powered by Editorial Manager® and ProduXion Manager® from Aries Systems Corporation

sure it is accurate.<br><br>**Unfunded studies**<br>Enter: *The author(s) received no specific funding for this work.*<br><br>**Funded studies**<br>Enter a statement with the following details:<br>• Initials of the authors who received each award<br>• Grant numbers awarded to each author<br>• The full name of each funder<br>• URL of each funder website<br>• Did the sponsors or funders play any role in the study design, data collection and analysis, decision to publish, or preparation of the manuscript?<br>• **NO** - Include this sentence at the end of your statement: *The funders had no role in study design, data collection and analysis, decision to publish, or preparation of the manuscript.*<br>• **YES** - Specify the role(s) played.<br><br>* typeset	
**Competing Interests**<br><br>Use the instructions below to enter a competing interest statement for this submission. On behalf of all authors, disclose any competing interests that could be perceived to bias this work—acknowledging all financial support and any other relevant financial or non-financial competing interests.<br><br>This statement is required for submission and **will appear in the published article** if the submission is accepted. Please make sure it is accurate and that any funding sources listed in your Funding Information later in the submission form are also declared in your Financial Disclosure statement.<br><br>View published research articles from *PLOS ONE* for specific examples.	The authors have declared that no competing interests exist.

Powered by Editorial Manager® and ProduXion Manager® from Aries Systems Corporation

**NO authors have competing interests**<br><br>Enter: *The authors have declared that no competing interests exist.*<br><br>**Authors with competing interests**<br><br>Enter competing interest details beginning with this statement:<br><br>*I have read the journal's policy and the authors of this manuscript have the following competing interests: [insert competing interests here]*<br><br>* typeset	
**Ethics Statement**<br><br>Enter an ethics statement for this submission. This statement is required if the study involved:<br><br>• Human participants<br>• Human specimens or tissue<br>• Vertebrate animals or cephalopods<br>• Vertebrate embryos or tissues<br>• Field research<br><br>Write "N/A" if the submission does not require an ethics statement.<br><br>General guidance is provided below. Consult the submission guidelines for detailed instructions. **Make sure that all information entered here is included in the Methods section of the manuscript.**	no

**Format for specific study types**<br><br>**Human Subject Research (involving human participants and/or tissue)**<br>• Give the name of the institutional review board or ethics committee that approved the study<br>• Include the approval number and/or a statement indicating approval of this research<br>• Indicate the form of consent obtained (written/oral) or the reason that consent was not obtained (e.g. the data were analyzed anonymously)<br><br>**Animal Research (involving vertebrate animals, embryos or tissues)**<br>• Provide the name of the Institutional Animal Care and Use Committee (IACUC) or other relevant ethics board that reviewed the study protocol, and indicate whether they approved this research or granted a formal waiver of ethical approval<br>• Include an approval number if one was obtained<br>• If the study involved *non-human primates*, add *additional details* about animal welfare and steps taken to ameliorate suffering<br>• If anesthesia, euthanasia, or any kind of animal sacrifice is part of the study, include briefly which substances and/or methods were applied<br><br>**Field Research**<br><br>Include the following details if this study involves the collection of plant, animal, or other materials from a natural setting:<br>• Field permit number<br>• Name of the institution or relevant body that granted permission	
**Data Availability**<br><br>Authors are required to make all data underlying the findings described fully available, without restriction, and from the time of publication. PLOS allows rare exceptions to address legal and ethical concerns. See the PLOS Data Policy and FAQ for detailed information.	No - some restrictions will apply

A Data Availability Statement describing where the data can be found is required at submission. Your answers to this question constitute the Data Availability Statement and **will be published in the article**, if accepted.<br><br>**Important:** Stating 'data available on request from the author' is not sufficient. If your data are only available upon request, select 'No' for the first question and explain your exceptional situation in the text box.<br><br>Do the authors confirm that all data underlying the findings described in their manuscript are fully available without restriction?	
**Describe where the data may be found in full sentences. If you are copying our sample text, replace any instances of XXX with the appropriate details.**<br><br>• If the data are **held or will be held in a public repository**, include URLs, accession numbers or DOIs. If this information will only be available after acceptance, indicate this by ticking the box below. For example: *All XXX files are available from the XXX database (accession number(s) XXX, XXX.*).<br>• If the data are all contained **within the manuscript and/or Supporting Information files**, enter the following: *All relevant data are within the manuscript and its Supporting Information files.*<br>• If neither of these applies but you are able to provide **details of access elsewhere**, with or without limitations, please do so. For example:<br><br>*Data cannot be shared publicly because of [XXX]. Data are available from the XXX Institutional Data Access / Ethics Committee (contact via XXX) for researchers who meet the criteria for access to confidential data.*<br><br>*The data underlying the results presented in the study are available from (include the name of the third party*	All data are available from the CSMAR database(https://www.gtarsc.com/)

Powered by Editorial Manager® and ProduXion Manager® from Aries Systems Corporation

and contact information or URL).<br>• This text is appropriate if the data are owned by a third party and authors do not have permission to share the data.<br><br>* typeset	
Additional data availability information:	

Click here to access/download;Manuscript;supply network_20220703231655.pdf

Supply network position, digital transformation and innovation performance: evidence from listed Chinese manufacturing firms

Chunyan Du[1,2]*, Qiang Zhang[1]

1 Business School, Yunnan University of Finance and Economics, Kunming, Yunnan, China
2 Financial Planning Department, China West Normal University, Nanchong, Sichuan, China

* candy4022@hotmail.com

Abstract

This study provides evidence for the supply network position influencing innovation performance and the moderating effect of digital transformation. Supply chain relationships have been evaluated in earlier research to demonstrate how concentrations of customers and suppliers may either favorably or adversely impact innovation. These metrics, however, only take into account how closely a firm is connected to its direct customers or suppliers. This study integrates the top five suppliers and customers of Chinese listed manufacturing firms and considers the relationship embeddedness of each firm's direct customers and suppliers, as well as the structure embeddedness among the customers' customers, customers' suppliers, suppliers' customers, and suppliers' suppliers to reveal the true impact of supply chain relationships on innovation performance.The top five suppliers and consumers of each firm are chosen to build a supply network for each year using panel data of listed Chinese manufacturing firms from 2013 to 2020. Social network analysis is used to determine network centrality and structural holes.The results show that in the supply network, network centrality and structural holes are significantly negatively correlated with innovation performance, especially in large firms, non-state-owned firms and firms in recession phase. According to the moderating effect model, digital transformation is an efficient way for declining the negative effect of supply network position on innovation performance. The research results will further improve the supply network cooperation mechanism, which is of great significance for improving supply chain resilience and firms' innovation.

1. Introduction

A key factor in regional economic development and a crucial component of China's competitiveness in science and technology is the high-quality innovation output of firms. Firms fully utilize external information and understand that knowledge transfer is crucial to enhancing innovation performance [1-2]. Resource dependency theory stresses that organizations must connect with other organizations to receive essential external resources, which creates the dependence on external resources, and that they cannot only rely on internal resources for existence. Organizations can access and sustain external resources through cooperative partnerships with other organizations when faced with the unpredictability of resource acquisition and organizational reliance[3]. Supply chain relationships bring opportunities for sustainable innovation of firms. ~~Supply chain relationships~~ originate from frequent contacts between suppliers and customers. In such relationships, the interaction between them forms a complex supply network. Many existing studies use supplier and customer concentration as a measure of supply chain relationships to prove that customer concentration positively [4-8] or negatively [9-11] affects innovation, or that supplier concentration negatively affects firms' innovation

[4,8,12-15]. However, these two indicators only reflect the relationship embeddedness between the firm and its direct suppliers or customers; they do not express the structure embeddedness between the firm and customers' customers, customers' suppliers, suppliers' customers, and suppliers' suppliers. Relevant research has examined the linear supply chain relationship but failed to build the network-level supply chain relationship; therefore, the effect of supply chain relationships on innovation performance is not revealed.

The contributions of this study are as follows: (1) This study analyzes the effect of firms' network locations on innovation performance by constructing a large sample of supply chain network. The findings of the study complement the existing literature and expand the application of social network theory in the supply chain field. (2) Existing research posits that firms in centrality and structural holes positions have information, resources, and control benefits that promote their profits [16-17]. However, this study conducts an empirical analysis which reveals that firms in supply network positions would level down innovation performance.

In this study, a research framework on the effect of supply network position on firms' innovation performance is established. The paper is structured as follows: Section 2 reviews the literature on innovation performance and supply networks at home and abroad; Section 3 presents the research hypotheses, model specification, and variables settings; Section 4 discusses the results from the basic regression model, which is followed by a moderating effect analysis; and finally, the main conclusions, limitations, and policy implications of this study are highlighted in Section 5.

2. Literature Review

Most of the existing studies exploring the factors affecting firms' innovation are based on the theory of endogenous growth that considers the scale of firms [18-20], ownership [21-22], corporate governance [23-24], and organizational culture [25-26] as important factors affecting innovation performance. The resource dependence theory suggests that different firms hold heterogeneous resources, which are usually scarce and difficult to imitate [27]. It is impossible for a firm to master all resources for innovation; therefore, firms must exchange knowledge and resources concerning innovation with other firms. Innovation can be realized through the external network embedded with knowledge and information resources [28]. Suppliers and customers in the supply chain can offer external innovation resources that the firms need. The sharing of knowledge and information between firms and supply chain partners is important for successful innovation [29]. The embeddedness theory posits that economic behavior is embedded in the social structure [30], and the core of a social structure is the social network. Thus, the activities and decision-making of firms are inevitably affected by the social network in which they are positioned. As one of the branches of the social network, the supply chain network has a profound impact on firms' development. Choi et al. (2001) first proposed that the supply chain relationship should be regarded as a complex network of connections, and suppliers and customers should be regarded as actors in the network[31]. There are a few empirical studies on supply networks; research has mainly focused on constructing supply networks for a single sub-sector industries. Choi and Hong (2002) characterized the supply network of the US auto industry in a case study[32]. Dong et al. (2015) studied the impact of relation and structure embeddedness on firms' opportunistic behavior by constructing a supply network in China's auto industry with the help of questionnaires and interviews[28]. Bellamy et al. (2014) selected 151 electronic companies from the top 300 companies in the world from 2007 to 2008 to construct a supply network and studied the effects of network accessibility and network interconnectivity on innovation ability[33]. Li et al. (2020) utilized the data of the electronics manufacturing industry of 200 listed firms in China from 2013 to 2017 and found that the centrality and structural holes in supply network positioning is significantly positively correlated with innovation performance. Few other studies have constructed supply networks based on multi-industry data[34]. Li et al. (2015) conducted a questionnaire survey of 237 manufacturing firms and observed that suppliers in network positions significantly promoted innovation performance[35]. Kim (2017) built a customer network based on 717 listed firms' information about their major customers from the Compustat database and discussed the impact of the interconnection of customers on firms' profitability[36]. Kao et al. (2017) built a supply network by

integrating the main customer information of US listed firms from 2005 to 2012 and analyzed the influence of supply network structure on the firms' productive efficiency[37]. Shi et al. (2019) constructed a supply chain network based on the names of the top five suppliers and customers of China's listed manufacturing firms from 2013 to 2016 and found that centrality and structural hole positions led to operational risk, which negatively affected the firms' performance[38]. Chen et al. (2021) collected the names of the suppliers and customers of China's top five listed manufacturing firms from 2014 to 2018 to form a supply network and found that the centrality position benefited innovation performance, while the structural hole position had a significant negative effect on innovation performance[39]. Yu et al. (2022) also constructed a supply chain network using the names of the suppliers and customers of China's top five listed firms from 2004 to 2019 and found that listed firms in a centrality position promoted firms' competitiveness[40].

Prior research has primarily used case studies or questionnaire surveys pertaining to supply chain networks and paid attention to specific industries. However, these studies lack empirical analyses using large sample data, although few studies have examined the effect of supply chain network position on innovation performance. Therefore, this study explores the relationship between supply network position and innovation performance, and discusses the moderating effect of digital transformation.

3. Research Design

3.1. Research Hypotheses

The behavior of actors in social and economic activities embedded in an interactive network is affected by the social network [30, 41]. The social network emphasizes its information, resource, and control benefits through relationship embeddedness and structure embeddedness. In the supply network, relationship embeddedness is manifested as repeated cooperation among network members, which can enhance trust and strengthen information sharing and resource complementarity. Existing studies have utilized customer and supplier concentrations to characterize relationship embeddedness [42-43, 9]. We focus on the effect of structural embeddedness in the supply network on innovation performance. Structure embeddedness emphasizes the informational roles that members occupy in the overall network [44], reflecting the aspect of information sharing resulting from interactions among members [45-46]. Network centrality and structural holes characterize the structure positions occupied by firms in a network. Therefore, we explore the effect of supply network centrality and structural holes on innovation performance.

3.1.1 Centrality and innovation performance

Network centrality is often used to evaluate the status and social prestige of members in social networks [47]. The higher the network centrality of a firm, the greater the number of partners of the firm. Firms occupying a centrality position in a social network have certain resources, information, and control benefits [48]. As they conduct more direct or indirect businesses with other firms in the network, it is easier for them to strengthen transfer of resources and acquire more advanced technology and knowledge, which contribute to improving innovation capabilities. These firms have many channels through which they can collect rich information to reduce transaction costs and enhance innovation capabilities [40]. Firms with higher centrality have more cooperation choice. This asymmetry of choice determines that firms with higher network centrality have stronger bargaining power in supply chain relationships [49-50], thereby improving their own profitability and innovation capabilities [51].
H1: There is a positive correlation between network centrality and innovation performance in a supply network.

3.1.2 Structural holes and innovation performance

Let us assume that firms A, B, and C exist in a network. A has direct connections with B and C in the social network, while B has no connections with C. The missing link between B and C indicates that there is a hole in the network structure[52]. According to the structural hole theory, a firm occupying a “bridge” position in the supply network can build a platform for communication between firms without direct business relationships. The greater the number of disconnected firms it connects, the greater the number of structural holes. Firms that are not directly connected in the network belong to different circles; each circle has its own unique information on technology and product price. Firms occupying “bridge” positions can obtain information from other circles. In a supply network, more structural holes could help firms access more valuable, non-redundant resources from multiple partners [53] and thereby grow diversified technology and knowledge. This would be the incentive for firms to invent new technological breakthroughs. Kim (2017) found that structural holes brought more heterogeneity and complementary knowledge to firms and were conducive to improving technological innovation[36]. Concurrently, firms in a structural hole position could have strong bargaining power in the transaction process through the information and control benefits in the supply network. Bargaining power not only directly determines firms’ profits, which could reduce operational risks with the help of sufficient innovation funds, but also makes it possible to avoid yielding to the claims of major customers, such as reducing sales prices, offering more commercial credit [54], reducing costs related to innovative activities.
H2: There is a positive correlation between structural hole position and innovation performance in a supply network.

3.1.3The moderating effect of digital transformation

As for the supply network, digital transformation will optimize the rationale for supply chain structure by improving the sensitive response to the market. The firms can timely identify the dynamic and diverse needs of consumers and potential suppliers through big data analysis. In order to keep the resilience of the supply network, the firms would revise their position and strengthen network structural adjustments to meet the value creation. Meanwhile, digital transformation realizes the diversity of the supply network. Digital technology speeds up the exchange of internal and external information [55] , which stimulates more cooperation in the supply chain network. In the practice of digital transformation, the supply chain network has created the cross-space virtual cooperation, effectively breaking the limitations of traditional supply chain integration. In addition, the application of advanced digital technologies help monitor members in the supply network [56] and utilize their resources scientifically.
H3:Digital transformation moderates the relationship between supply network position and innovation performance.

3.2. Model Specification and Variable Setting

The following models are constructed:

Basic model: $\ln IP_{it} = \alpha_0 + \alpha_1 X_{it} + \alpha_2 control_{it} + \sum Industry + \sum Year + \varepsilon_{it}$

Moderating model: $\ln IP_{it} = \theta_0 + \theta_1 X_{it} + \theta_2 \ln DT_{it} + \theta_3 X_{it} \times \ln DT_{it} + \theta_4 control_{it} + \sum Industry + \sum Year + \varepsilon_{it}$

Here, IP_{it} refers to the innovation performance of the i_{th} firm in year t; X_{it} is the network position of the i_{th} firm in the t_{th} year; DT_{it} represent digital transformation of the i_{th} firm in year t; controlit denotes a vector consisting of a series of control variables involving Lev, Grow, Share, Fixed_assets and Roa. Industry and Year are dummy variables. It is necessary to consider each model coefficient and its significance, as this aids in investigating the relationship between supply network position and innovation performance.
The explained variable, innovation performance (IP), can be measured by the number of patent applications. In the model, we apply the logarithm of the number of patent applications to the research.
The explanatory variable in (1), that is the network position (X), can be expressed by the centrality and structural holes. The centrality of nodes in the network mainly includes

degree centrality, betweenness centrality, eigenvector centrality, and closeness centrality. Degree centrality refers to the number of firms in the network that are directly connected, which reflects the degree of control over the resources of core firms in the network [57]. It is presented in formula (1).

$$DC(n_i) = \sum_{i=1}^{n} x(p_i p_j) \quad (1)$$

Here, n is the number of network nodes. If nodes i and j connect, x(pipj)=1; otherwise, x(pipj)=0.

Betweenness centrality measures the role of a firm acting as a “bridge” between other firms.

Eigenvector centrality is measured by considering the degree centrality of other firms connected in the network. Closeness centrality measures the degree of independence of firms in obtaining information and resources in a connected network. Since the supply network is not fully connected, closeness centrality is not suitable for our analysis. This study adopts degree centrality (DC) as the measurement scale for network centrality position.

Structural holes (SH) are utilized to measure the redundancy of resources in the network [52]. If two firms establish a connection with a third firm simultaneously, but there is no direct connection between the two firms, the third firm is considered to be in a structural hole position [58].

It can be calculated using formula (2).

$$SH(n_i) = 1 - \sum_{j} (p_{ij} + \sum_{q} p_{iq} p_{qj})^2 \quad (2)$$

Here, pij is the proportion of the direct connections between nodes i and j in all the connections of i in the network; piq and pqj are the proportion of the indirect connections between nodes i and j in all the connections of i in the network.

DT, can be indicated by specific keywords in the annual reports of listed manufacturing firms, like artificial intelligence, big data, blockchain, cloud computing and intelligent manufacturing. We search these specific keywords using Python and utilize the final total word frequency to measure the degree of digital transformation of a firm.

The following control variables are considered:

(1) Lev is the ratio of a firm’s total liabilities to total assets.

(2) Grow, measured by the firm's net profit growth rate, is obtained from the Tobin’s Q value.

(3) Share is measured using the shareholding ratio of the firm's largest shareholder.

(4) Fixed_assets are stated by the ratio of fixed assets to total assets.

(5) Roa is the ratio of net profit to average total assets.

3.3. Sample Selection and Data Description

“Standards on the Contents and Formats of Information Disclosure by Firms Offering Securities to the Public No. 2 - Contents and Formats of Annual Reports” was published by the China Securities Regulatory Commission in 2012. It encourages listed firms to record the names of their top five suppliers and customers. Therefore, this study collects the information of the top five suppliers and customers and other financial data of listed manufacturing firms from 2013 to 2020 from the CSMAR database. If one of the suppliers or customers is a listed firm, we continue to search the information on its top five suppliers and customers. After we had eliminated the samples with missing information, data of 3403 firms were finally obtained. Social network analysis is the process of investigating social structures through the use of networks and graph theory. It characterizes networked structures in terms of nodes (actors or things within the network) and the ties, edges, or links (relationships or interactions) that connect them. Social network analysis is the application of network science on social networks, and graphs are convenient

mathematical representations, considered as the basis of this study. We construct the supply network with a one-mode matrix and calculate centrality and structural holes using the Ucinet software.

Descriptive statistics and correlation analysis of variables are conducted in the supply network using the STATA software (Table 1). A multicollinearity analysis is also conducted; the results show that the maximum variance inflation factor (VIF) is 3.62, reflecting that there is no multicollinearity between the variables.

Table 1 Descriptive statistics of each variable

Variable	Obs	Mean	Std.Dev.	Min	Max
lnIP	3,403	2.0954	1.8142	0	9.4733
DC	3,403	0.2048	0.1215	0.0200	0.4480
SH	3,403	0.6849	0.3493	-0.1250	0.9180
Lev	3,403	0.5148	3.1047	0.0080	178.3455
Roa	3,403	0.0076	0.7139	-30.9587	1.1261
Share	3,403	33.0076	14.8734	3.8900	89.9900
Grow	3,296	2.1776	2.5804	0.6992	106.1318
Fixed_ass	3,403	0.2380	0.1548	0	0.8098
lnDT	3,403	1.0299	1.1881	0	5.7366

4. Empirical Results and Discussion

4.1. Hypothesis Test

Table 2 shows the regression results of the effect of supply network position on innovation performance. As indicated, network centrality affects innovation performance negatively, and the coefficient of degree centrality is -3.960 ($p<0.01$) in Model I-1. Structural holes affect innovation performance significantly at the 1% level, and the coefficient is still negative (-1.337) in Model I-2. The empirical results strongly disagree with H1 and H2. The result is still likely to be endogenous because the causality would not be confirmed or important variables maybe omitted. Thus, the explanatory variables are lagged one year to get Model II, which indicating that there is no serious endogenous problem in the basic regression model. The results demonstrate that supply network position has a notably negative influence on innovation performance. With an increase of centrality and structure holes, the firm does not obtain resources, information, and control benefits, which is unfavorable for innovation performance. The reason may be that the network position would benefit the firms based on the assumption that resource sharing and information transmission of each firm in the social network is absolutely effective. As far as the central position of the supply network is concerned, even if the firm can contact other firms by its core position, which has more channels for obtaining resources and information, a low degree of marketization makes it hard to facilitate the free flow of information and resources among firms. Although firms in structural holes can theoretically obtain more heterogeneous external information, information asymmetry among firms also makes it difficult to establish mutual trust and promote effective transmission, thus weakening the firm's innovation ability.

Table 2 Results of regression models

	I-1	I-2	II-1	II-2
	lnIP	lnIP	lnIP	lnIP
DC	-3.960***			
	(0.247)			
SH		-1.337***		
		(0.0825)		
L.DC			-4.517***	
			(0.355)	
L.SH				-1.511***
				(0.121)
Lev	0.0872***	0.0859***	0.0842***	0.0816***
	(0.0160)	(0.0160)	(0.0169)	(0.0169)
Grow	-0.117***	-0.112***	-0.120***	-0.116***
	(0.0158)	(0.0158)	(0.0179)	(0.0180)
Share	0.0114***	0.0116***	0.0157***	0.0154***
	(0.002)	(0.002)	(0.0027)	(0.0027)
Fixed_ass	-0.514**	-0.500**	-0.552*	-0.534*
	(0.220)	(0.219)	(0.298)	(0.298)
Roa	0.121*	0.131**	0.0798	0.0843
	(0.0636)	(0.0636)	(0.0761)	(0.0762)
cons	2.886***	3.356***	3.003***	3.431***
	(0.476)	(0.478)	(0.655)	(0.658)
Industry	Y	Y	Y	Y
Year	Y	Y	Y	Y
N	3296	3296	1913	1913
R2	0.3046	0.3056	0.3430	0.3410

4.2. Robustness Test

A robustness test is also performed to increase the credibility of the results. There are numerous methods for testing robustness, such as alternative variables, supplementary variables, sub-sample regression, model substitution, and changing the sample size. For the robustness test, we replace the number of patent applications, degree centrality, and structural holes in the model with R&D investment, eigenvector centrality [39], and betweenness centrality [59], respectively. The relevant empirical results are shown in Table 3. The direction of each variable is consistent with Model I. The coefficients of degree centrality and structural holes in Models III, VI, and V are different from one another. Hence, the results of Model I are robust.

Table 3 Results of the robustness test

	III-1	III-2	IV	V
	lnRD	lnRD	lnIP	lnIP
DC	-3.219***			
	(0.236)			
SH		-1.046***		
		(0.0797)		
EC			-0.581*	
			(0.325)	
BC				-0.0042***
				(0.0007)
Lev	2.004***	1.981***	0.100***	0.100***
	(0.143)	(0.143)	(0.0166)	(0.0165)
Grow	-0.197***	-0.194***	-0.126***	-0.129***
	(0.0192)	(0.0193)	(0.0164)	(0.0163)
Share	0.0165***	0.0168***	0.0134***	0.0138***
	(0.0019)	(0.0019)	(0.0021)	(0.0021)
Fixed_ass	-0.568***	-0.546**	-0.412*	-0.424*
	(0.220)	(0.220)	(0.228)	(0.227)
Roa	2.000***	1.987***	0.181***	0.174***
	(0.155)	(0.156)	(0.0660)	(0.0656)
cons	16.79***	17.17***	2.778***	2.785***
	(0.468)	(0.471)	(0.495)	(0.492)
Industry	Y	Y	Y	Y
Year	Y	Y	Y	Y
N	3000	3000	3296	3296
R2	0.3858	0.3832	0.2498	0.2583

4.3. Heterogeneity Analysis

The effect of supply network position on innovation across various firm sizes is seen in Table 4. The firms are categorized into large firms (Model VI) and small and medium-sized ones based on the mean value of the total assets (Model VII). Large firms can draw in more supplier network partners since they have stronger financial strength than small and medium-sized firms. The relationship inside the network is more complicated, and there is a greater likelihood that they may become entangled by the terms of the cooperative partners' operations. Therefore, the centrality of large firms has a greater detrimental effect on innovation performance than that of small and medium-sized firms because the fragility of the network may result in bankruptcy or worse efficiency[60]. Weisbuch and Battiston (2007) confirmed that partial failures in the supply network would lead to a rapid shortage of the entire networks' products, resulting in some firms going bankrupt in a simulation analysis[61]. The losses brought on by opportunistic behavior will also be bigger than those of small and medium-sized firms, and the more structural holes large firms have, the less they can control their partners. Therefore, compared to small and medium-sized firms, large firms are more sensitive to the performance of innovation due to network centrality and structural holes.

Table 4 The effect of supply network position on innovation under different firm sizes

	VI-1	VI-2	VII-1	VII-2
	lnIP	lnIP	lnIP	lnIP
DC	-3.984***		-1.921***	
	(0.414)		(0.305)	
SH		-1.109***		-0.787***
		(0.132)		(0.109)
Lev	1.462***	1.540***	0.0684***	0.0677***
	(0.347)	(0.349)	(0.0137)	(0.0137)
Grow	0.0130	0.0177	-0.104***	-0.101***
	(0.0505)	(0.0509)	(0.0148)	(0.0148)
Share	0.0113***	0.0120***	0.00419*	0.00402*
	(0.00332)	(0.00334)	(0.00233)	(0.00233)
Fixed_as s	0.102	0.0575	-0.685***	-0.646***
	(0.415)	(0.419)	(0.237)	(0.236)
Roa	5.150***	5.324***	0.0715	0.0748
	(0.956)	(0.964)	(0.0520)	(0.0517)
_cons	1.846**	2.113***	1.629**	2.001***
	(0.733)	(0.743)	(0.636)	(0.638)
Industry	Y	Y	Y	Y
Year	Y	Y	Y	Y
N	1389	1389	1907	1907
R2	0.3827	0.3730	0.2136	0.2188

Table 5 details the relationship between supply network position and innovation in state-owned and non-state-owned firms. State-owned firms (Model VIII) may be more prominent, which may explain why the centrality and structural holes of non-state-owned firms (Model IX) have a bigger detrimental effect on innovation than state-owned firms (Model VIII). State authority safeguards the development of state-owned businesses despite the fact that their organizational inertia results in significant dependence and a constrained flow of resources. Costs, benefits, and network position do not determine how innovative state-owned firms are. Non-state-owned firms are more likely to be impacted by the supply network while having higher operational risk and financial costs. The numerous structural flaws in non-state-owned firms make it impossible to effectively stop partners from behaving opportunistically, and the absence of a trust-building mechanism will lead to unstable cooperative partnerships. This hurts firms' ability to tap outside innovative resources.

Table 5 The effect of supply network position on innovation in state-owned and non-state-owned firms

	VIII-1	VIII-2	IX-1	IX-2
	lnIP	lnIP	lnIP	lnIP
DC	-3.624***		-3.805***	
	(0.436)		(0.304)	
SH		-1.093***		-1.351***
		(0.141)		(0.102)
Lev	1.151***	1.190***	0.0603***	0.0584***
	(0.262)	(0.263)	(0.0158)	(0.0157)
Grow	-0.118***	-0.114***	-0.0825***	-0.0763***
	(0.0419)	(0.0420)	(0.0169)	(0.0168)
Share	0.00251	0.00352	0.0140***	0.0138***
	(0.0036)	(0.0036)	(0.0025)	(0.0024)
Fixed_ass	0.149	0.130	-0.755***	-0.754***
	(0.399)	(0.400)	(0.277)	(0.276)
Roa	1.830***	1.827***	0.0933	0.101*
	(0.576)	(0.579)	(0.0602)	(0.0600)
_cons	1.877	2.291**	2.988***	3.454***
	(1.145)	(1.155)	(0.502)	(0.502)
Industry	Y	Y	Y	Y
Year	Y	Y	Y	Y
N	1184	1184	2026	2026
R2	0.4444	0.4403	0.2134	0.2199

The firm's life cycle can be separated into three phases: growth(Model X), maturity (Model XI), and recession (Model XII)[62]. Table 6 displays the effect of supply network position on innovation at various stages. We observe that network position has a detrimental impact on innovation in all phases, with the effects during the recession being the most pronounced. This could be as a result of the fact that firms in the growth phase have high learning and absorptive skills to gather non-redundant and heterogeneous knowledge to drive expansion, whereas firms in the maturity phase have great resource accumulation. Some of the detrimental effects of operational risk brought on by network instability can be mitigated by the strong innovation pace in the two phases. On the other hand, firms in the recession phase have no resources and control advantages, and their funds are more used to maintain basic survival, and it is more difficult to find breakthroughs in innovation.

Table 6 The effect of supply network position on innovation at various stages

	X-1	X-2	XI-1	XI-2	XII-1	XII-2
	lnIP	lnIP	lnIP	lnIP	lnIP	lnIP
DC	-2.954***		-4.025***		-5.018***	
	(0.363)		(0.411)		(0.633)	
SH		-1.073***		-1.280***		-1.635***
		(0.121)		(0.137)		(0.219)
Lev	1.247***	1.195***	1.407***	1.359***	0.224***	0.237***
	(0.235)	(0.235)	(0.239)	(0.241)	(0.0598)	(0.0600)
Grow	-0.104***	-0.0979***	-0.0702**	-0.0660**	-0.0754***	-0.0754***
	(0.0319)	(0.0318)	(0.0281)	(0.0282)	(0.0239)	(0.0241)
Share	0.00653**	0.00626**	0.00600*	0.00718**	0.0243***	0.0233***
	(0.0029)	(0.0029)	(0.0032)	(0.0032)	(0.0049)	(0.0049)
Fixed_ass	-0.690**	-0.664*	-0.806**	-0.845**	0.880*	1.036**
	(0.344)	(0.342)	(0.358)	(0.359)	(0.520)	(0.524)
Roa	1.234***	1.191***	3.667***	3.659***	1.182***	1.259***
	(0.248)	(0.247)	(0.551)	(0.554)	(0.379)	(0.381)
_cons	2.588***	3.097***	2.032***	2.398***	-2.523*	-2.059
	(0.652)	(0.655)	(0.725)	(0.730)	(1.436)	(1.446)
Industry	Y	Y	Y	Y	Y	Y
Year	Y	Y	Y	Y	Y	Y
N	1479	1479	1317	1317	500	500
R2	0.2740	0.2804	0.3720	0.3680	0.3567	0.3474

4.4. Moderating Effect

It can be seen that digital transformation significantly positively affects innovation performance significantly from Table 7, that is; a 1% rise of digital transformation can increase the firms' innovation by 0.513% and 0.469%. The coefficient of digital transformation × degree centrality(-1.341) and digital transformation × structural holes(-0.362) are smaller than that of degree centrality (-2.268) and structural holes(-0.858) at the 1% level in Model XIII, which reflect that digital transformation weakens the adverse effects of degree centrality and structural holes on innovation in the firms significantly. The empirical results strongly support H3. Digital transformation is efficient for moderating the impact of supply network position on innovation performance.

Table 7 Results of the moderating effect of digital transformation

	XIII-1	XIII-2
	lnPI	lnPI
DC	-2.268***	
	(0.327)	
SH		-0.858***
		(0.111)
lnDT	0.513***	0.469***
	(0.0477)	(0.0500)
DC*lnDT	-1.341***	
	(0.186)	
SH*lnDT		-0.362***
		(0.0628)
Lev	0.0829***	0.0816***
	(0.0157)	(0.0157)
Grow	-0.111***	-0.106***
	(0.0155)	(0.0155)
Share	0.0097***	0.0104***
	(0.002)	(0.002)
Fixed_ass	-0.197	-0.192
	(0.220)	(0.220)
Roa	0.119*	0.129**
	(0.0625)	(0.0626)
_cons	2.181***	2.609***
	(0.472)	(0.477)
Industry	Y	Y
Year	Y	Y
N	3296	3296
R2	0.3298	0.3264

5. Conclusions and Policy Implications

This study integrates the data from top five suppliers and customers of Chinese listed manufacturing firms and considers the relationship embeddedness of each firm's direct customers and suppliers, as well as the structure embeddedness among the customers' customers, customers' suppliers, suppliers' customers, and suppliers' suppliers. We construct a supply network of manufacturing firms with the data from 2013 to 2020 through social network analysis and measure the effect of supply network position on innovation performance. The research conclusions are as follows. First, the degree centrality and structural holes of the supply network did not promote innovation performance. Results suggest that in the supply network, the resources, information, and control advantages of degree centrality and structural holes have not been effectively exerted. This effect is more obvious in large firms,non-state-owned firms and firms in recession phase. Second, digital transformation is a efficient way for declining the negative effect of supply network position on innovation performance.

This study has limitations it is important for future research to address. First, there is scope for a follow-on study on the effect of supply network position on innovation

performance in some other industries (such as energy and agriculture), which may have unexpected results and findings. Second, suppliers' and customers' information about non-listed firms could be adopted to further extend the established network. Third, the nonlinear relationship or mediation effect could be explored to understand more clearly how they interact with each other. All of these could create more breakthroughs for suggestions in the next step.

The findings also have some policy implications. First, firms should maintain a harmonious trading environment and actively create an efficient supply network. The supply network may lack resilience when one firm in the network is in crisis, and other firms are likely to be affected. This suggests that firms embedded in the same supply network should jointly maintain a harmonious and orderly trading environment, resist market opportunism, and promote mutual trust and cooperation with their partners to reduce risks. Second, optimizing external environment benefits fosters innovation in a supply network. Firms located in the center of the supply network and occupying more structural holes face greater risks and financial constraints, reducing their willingness to innovate. Therefore, firms should stay in an adequate position in the supply network, appropriately increase the concentration of suppliers and customers, and select a few firms for stable business cooperation to avoid cooperating with a large number of firms with higher operational risks. Moreover, firms should focus on establishing strong ties with other firms and establish strategic alliances with supply network members to govern vicious market competition. This prevents opportunistic behavior by partners and reduces risk. Third, firms should formulate long-term plans for digital strategies considering their own network position, making up for the shortage of resources through the construction of basic digital capabilities, paying special attention to combining digital strategies with the current market and technology.

Author Contributions

Conceptualization: Qiang Zhang.
Data curation: Chunyan Du.
Formal analysis: Chunyan Du.
Methodology: Chunyan Du.
Project administration: Qiang Zhang.
Resources: Chunyan Du.
Supervision: Qiang Zhang.
Writing – original draft: Chunyan Du.
Writing – review & editing: Qiang Zhang, Chunyan Du.

References

1. Cohen WM, Levinthal DA. Absorptive capacity:a new perspective on learning and innovation. Administrative Science Quarterly. 1990;35:128-152.
2. Peng ZL, Wang HH, Jiang XC. Effect of resource sharing on innovation performance under the open innovation model:the mediating effect of knowledge transfer. Science of Science & Management of S&T. 2011;32(1):48-53.
3. Pfeffer J, Salancik GR. The external control of organizations: A resource dependence perspective. Cambridge:Stanford University Press; 1978.
4. Guo XL, Li K. Supply chain concentration, market status and R&D investment-research from horizontal and vertical perspectives. Industrial Economic Review. 2019;10(2):6-19.
5. Huang QY, Song YF. An empirical study on the impact of supply chain concentration on enterprises' R&D investment intensity-moderation role of ownership. R&D Management. 2019;31(3):13-26.
6. Ji L., Tao CJ. Can supply chain partners promote innovation performance based on the analysis of supplier and customer concentration. Journal of Zhongnan University of Economics and Law. 2019,1:38-46+65+159.
7. Wu C. Customer concentration and enterprise innovation: the effects of changes in supply chain finance. Financial Economics Research. 2021;36(5): 114-132.

8. Xu XM, Quan XF, Zhu SS. Supply chain concentration and firm innovation:empirical study based on listed manufacturing firms in China. Journal of Business Economics. 2022.4:5-16.
9. Meng QX, Bai J, Shi W. Customer concentration and enterprise technological innovation: help or hinder? a study based on the individual characteristics of customers. Nankai Business Review. 2018;21(4): 62-73.
10. Wan Q, Cheng XK, Wu CD. Customer concentration and enterprise innovation: empirical analysis of a share listed companies in China. Scientific Decision Making. 2017;10:22-48.
11. Zhang Y, Lan FY, Shen, HY. Customer concentration and company innovation-based on the perspective of customer bargaining power and property rights. China Business and Market.2019;33(4):76-88.
12. Han ZX, Duan LN, Gao XY. Supplier concentration and technological innovation-based on the adjustment of internal capital market and trade credit. Soft Science. 2021;35(9): 61-67.
13. Wang Y, Wang XL. Institutional environment,supplier concentration and enterprise R&D innovation output-empirical evidence from listed companies in China. Journal of Industrial Technological Economics. 2020;39(2):37-46.
14. Wang SN, Zhao S. Social trust, supplier relationship and firm innovation. Journal of Zhongnan University of Economics. 2020;6:25-34 .
15. Zhou MF, Zhang XD. Does a firm's supplier concentration affect its innovation. Review of Investment Studies. 2020;39(12):44-63.
16. Bagul A, Mukherjee I. An insight into centralized sourcing strategy for enhancing performance and sustainability of multi-tier supply network. International Journal of Intelligent Enterprise. 2017;5: 18-49.
17. Sha HW, Zeng Y. An empirical study on the cross-shareholding,network location and firm performance. Journal of Management Science. 2014;27(1):131-142.
18. Chi RY, Yu J, Ruan HP. Research on the influence of enterprise scale and R&D on innovation performance:based on the perspective of credit environment and knowledge stock. East China Economic Management. 2020; 34(9):43-54 .
19. Tang MP, Li HJ. Firm size, minimum wage and firm R&D investment. R&D Management. 2019;31(1): 44-55.
20. Yin XK, Zuscovitch E. Is firm size conducive to R&D choice? A strategic analysis of product and process innovations. Journal of Economic Behavior & Organization. 1998;35(2):243-262.
21. Gao G. The nature of property rights and innovation performance of enterprise. Commercial Research. 2013;5: 20-25.
22. Qian L, Wang WP, Xiao RQ. Ownership, technology gap and innovation efficiency of high-tech enterprises. Science & Technology Progress and Policy. 2019;36(12): 105-114.
23. Jia CX, Liu YJ. The influence of corporate governance structure on enterprise innovation performance-based on the intermediary role of R&D investment. Scientific Management Research. 2019; 37(2):117-121.
24. Li W, Bai YX, Wei SQ, Yu X. The influence of corporate governance on enterprise technological innovation-based on the perspective of industry analysis. Fujian Tribune. 2018;11: 49-58.
25. Gao CG, Xin J. The influence of corporate culture on the performance of independent innovation of enterprises: the mediating role of organizational learning ability. Dongyue Tribune. 2018;39(4): 68-75.
26. Zhao XY, Liu YZ. The impact of adaptive organizational culture on dynamic innovation capability and enterprise innovation performance. Soft Science. 2020;34(9): 79-84 .
27. Lv ZY. Resource dependence, network centrality and multi-party alliance construction: An empirical research of industry-sponsored e-marketplaces. Chinese Journal of Management. 2007;4: 509-513.
28. Dong MC, Liu Z, Yu Y, Zheng JH. Opportunism in distribution networks: the role of network embeddedness and dependence. Production & Operations Management. 2015;24(10):1657-1670.
29. Hobday M, Rush H, Tidd J. Innovation in complex products and system. Research Policy, 2000;29:793-804.

30. Granovetter M. Economic action and social structure:the problem of embeddedness. American Journal of Sociology. 1985;91(3): 481-510.
31. Choi TY, Dooley KJ, Rungtusanatham M. Supply networks and complex adaptive systems:control versus emergence. Journal of Operations Management. 2001;19(3):351-366.
32. Choi TY, Hong Y. Unveiling the structure of supply networks:case studies in Honda,Acura, and Daimler Chrysler. Journal of Operations Management.2002; 20(5): 469-493.
33. Bellamy, M. A.,Ghosh, S. and Hora, M. (2014), " The influence of supply network structure on firm innovation. " Journal of Operations Management, Vol.32 No.6, pp. 357-373.
34. Li GH, Zhao S, Wang Y.Supply network position, absorption capacity and firms' innovation performance. Soft Science. 2020;34(12): 1-7.
35. Li N, Li SC, Wang W. Supplier supply network position and manufacturer performance:The effects of network awareness capability. Journal of Management Science. 2015;28(2): 49-59.
36. Kim YH. The effects of major customer networks on supplier profitability. Journal of Supply Chain Management. 2017;53(1):26-40.
37. Kao TW, Simpson NC. Relating supply network structure to productive efficiency:A multi-stage empirical investigation. European Journal of Operational Research. 2016;259(2):469-485.
38. Shi JY, Yang JH, Li YX, Zhang XW. A slight move in one part may affect the situation as a whole: supply network location, operational risk and corporate performance", China Industrial Economics. 2019;9:136-154.
39. Chen L, Zhou ZB, Huang J. Location in supply network,management capability, and firm technological innovation base on financial and risk perspectives. Journal of Systems Science and Mathematical.2021; 41(11):3078-3092.
40. Yu MY, Lv KF, Ruan YP. Supply chain network location and enterprise competitive position. Systems Engineering-Theory & Practice. 2022;1-17.
41. Uzzi B. Social structure and competition in interfirm networks:The paradox of embeddedness. Administrative Science Quarterly. 1997;42(1):35-67.
42. Benlemlih M, Bitar M. Corporate social responsibility and investment efficiency. Journal of Business Ethics. 2018;148 (3): 647-671.
43. Irvine P, Park SS, Yildizhan, Celim. Customer-base concentration, profitability and the relationship life cycle. The Accounting Review. 2015;91(3): 883-906.
44. Gulati R. Alliances and Networks. Strategic Management Journal. 1998;19(4): 293-317.
45. Gulati R, Gargiulo M. Where do interorganizational networks come from. American Journal of Sociology. 1999;104(5):1439-1493.
46. Polidoro JF, Ahuja G, Mitchell W. When the social structure overshadows competitive incentives:The effects of network embeddedness on joint venture dissolution. Academy of Management Journal. 2011;54(1): 203-223.
47. Luo JD. Social network analysis handout. Beijing:Social Science Literature Press;2005.
48. Salman N, Saives AL. Indirect networks: an intangible resource for biotechnology innovation. R&D Management. 2005;35(2):203-215.
49. Cui V, Yang H, Vertinsky I. Attacking your partners: strategic alliances and competition between partners in product markets. Strategic Management Journal. 2018;39(12): 3116-3139.
50. Lavie D. Alliance portfolios and firm performance: A study of value creation and appropriation in the U.S. software industry. Strategic Management Journal. 2007;28(12): 1187-1212.
51. Suutari R. Understanding industry structure. CMA Management. 2000;73(11): 34-37.
52. Burt RS. Structural holes:the social structure of competition, Cambridge: Harvard University Press;2009 .
53. Mohammed B, Mohammad B. Corporate social responsibility and investment efficiency. Journal of Business Ethics. 2018;148(3): 647-671.

54. Li X, Yang JP. How alliance choice affects innovation performance-mediation based on structural holes. Science & Technology Progress and Policy. 2020;37(15): 80-88.
55. Nambisan S, Wright M，Feldman M. The digital transformation of innovation and entrepreneurship: progress, challenges and key themes. Research Policy. 2019;48(8): 1-9.
56. Ali Z, Bi GB, Mehreen A. Does supply chain finance improve SMEs performance? The moderating role of trade digitization. Business Process Management Journal. 2018;26(1):150-167.
57. Wei L, Dang XH. The inter-temporal coupling of routine and effect on iterative innovation:Moderated mediation effects. Studies in Science of Science. 2018;36(5): 933-945.
58. Zhang HJ, Tan JS. Alliance networks and firms' innovation performance: A cross-level analysis. Management World. 2014;3: 163-169.
59. Liu J. Network analysis lecture notes: A practical guide to UCINET software. Shanghai:Shanghai People's Press;2009 .
60. Rezapour S, Srinivasan R, Tew J, Allen JK, Mistree F. Correlation between strategic and operational risk mitigation strategies in supply networks. International journal of production economics. 2018;201(7):225-248.
61. Weisbuch G, Battiston S. From production networks to geographical economics. Journal of Economic Behavior & Organization. 2007;64(3-4): 448-469.
62. Huang HB, Zhai SP, Chen JN. Corporate life cycle,financing methods and financing constraints-based on the moderating effect research of investor sentiment. Journal of Financial Research. 2016;7:96-112.
