# Supplementary material for: Supply network position, digital transformation and innovation performance: Evidence from listed Chinese manufacturing firms
Source: PLoS One. 2022 Dec 15;17(12):e0279133. doi: 10.1371/journal.pone.0279133 (PMC9754206; doi:10.1371/journal.pone.0279133)
Supplement: Attachment — Submitted filename: Response to Reviewers.docx [file pone.0279133.s004.docx]

I'd like to thank the reviewer for his/her attentive reading, useful remarks, and constructive ideas, which have substantially enhanced the manuscript's presentation. I carefully studied the reviewer' remarks and updated my article accordingly.

Journal Requirements:

Please review your reference list to ensure that it is complete and correct. If you have cited papers that have been retracted, please include the rationale for doing so in the manuscript text, or remove these references and replace them with relevant current references. Any changes to the reference list should be mentioned in the rebuttal letter that accompanies your revised manuscript. If you need to cite a retracted article, indicate the article’s retracted status in the References list and also include a citation and full reference for the retraction notice.

*Response:*

I have updated the reference part carefully(page 13-16) in the revised manuscript.

Reviewer 1

I have made a few more suggestions in the text, mostly concerning the introduction and literature review section. Here, there are still some parts that are unclear and/or could be improved.

*Response:*

I have removed some sentences marked in the Introduction part (page2).

I have paraphrased some sentences and reorganized the reference order in the Literature Review (page 3-4).

In the **Descriptive Statistics and Analysis** part, spearman correlation and multicollinearity analysis are also performed. Even though a few values are relative highly correlated; the results reveal that the maximum variance inflation factor (VIF) in Model (1)-(4) is 2.55, 2.54, 1.86 and 1.86, reflecting that there is no multicollinearity between the variables. Some other errs are corrected in the revised copy with track changes.
